# Design of Belief Propagation Based on FPGA for the Multistereo CAFADIS Camera

**DOI:** 10.3390/s101009194

**Published:** 2010-10-15

**Authors:** Eduardo Magdaleno, Jonás Philipp Lüke, Manuel Rodríguez, José Manuel Rodríguez-Ramos

**Affiliations:** Departamento de Física Fundamental y Experimental, Electrónica y Sistemas, University of La Laguna, Avd. Francisco Sanchez s/n, 38203 La Laguna, Spain; E-Mails: jpluke@ull.es (J.P.L.); mrvalido@ull.es (M.R.); jmramos@ull.es (J.M.-R.)

**Keywords:** plenoptic sensors, FPGA, real-time processing, depth estimation, multistereo

## Abstract

In this paper we describe a fast, specialized hardware implementation of the belief propagation algorithm for the CAFADIS camera, a new plenoptic sensor patented by the University of La Laguna. This camera captures the lightfield of the scene and can be used to find out at which depth each pixel is in focus. The algorithm has been designed for FPGA devices using VHDL. We propose a parallel and pipeline architecture to implement the algorithm without external memory. Although the BRAM resources of the device increase considerably, we can maintain real-time restrictions by using extremely high-performance signal processing capability through parallelism and by accessing several memories simultaneously. The quantifying results with 16 bit precision have shown that performances are really close to the original Matlab programmed algorithm.

## 1. Introduction

3D reconstruction has been a very active research field for many years. The problem can be approached with active techniques, in which the system interacts with the scene, or with passive techniques in which the system, instead of interacting with the scene, captures images from several view points in order to reconstruct the scene-related depth information.

Using passive techniques, only two views are enough to reconstruct 3D information from the scene by means of a stereo algorithm. However, these techniques can be generalized to more than two views and are then called multistereo techniques. Both dual stereo and multistereo are generally based on finding a correspondence between the pixels of several images taken from different view points. This is called the correspondence problem and generally needs some optimization process in order to find the best correspondence between pixels.

The correspondence problem can be solved within the Markov Random Field (MRF) framework [1–3]. However, this yields an optimization problem that is NP-hard. Satisfactory techniques have been developed to find approximate solutions, namely graph cuts and belief propagation. These techniques are very demanding in computational terms, if compared to other techniques. Although these techniques produce good results, they are slow. This is an impediment when 3D reconstruction warrants real-time performance, for example in a 3DTV video camera.

CAFADIS is a 3D video camera patented by the University of La Laguna that performs depth reconstruction in real time. The CAFADIS camera is an intermediate sensor between the Shack-Hartmann and the pyramid sensor [4]. It uses a plenoptic camera configuration in order to capture multiview information (it samples an image plane using a microlens array) [4]. This multiple view information is composed of hundreds of images taken from slightly different points of view that are captured with a single lens, single body device. Plenoptic sensors capture the lightfield of the scene and can be used to synthesize a set of photographs focused at different depths in the scene [4–9]. The image resulting from application of the CAFADIS sensor can be seen as formed by four dimensions: two CCD co-ordinates associated to each microlens and a further two co-ordinates stemming from the microlens array. These can then be used to estimate a focus measure usable as cost function that has to be optimized in order to find out at which depth each pixel is in focus [10]. As a consequence, a depth value can be assigned to each pixel. This 3D map, combined with the 2D scene image, can be used as input for a 3D display. This optimization process can also be done within a MRF framework by means of the belief propagation algorithm.

The optimization process is very slow, so specific hardware has to be used to achieve real-time performance. A first prototype of the CAFADIS camera for 3D reconstruction was built using a computer provided with multiple Graphical Processing Units (GPUs) and achieving satisfactory results [4,10]. However, this hardware has the disadvantage of not being portable in the least. Now, the goal is to obtain full portability with a single lens, single body optical configuration and specific parallel hardware programmed on Field Programmable Gate Arrays (FPGAs).

The FPGA technology makes the sensor applications small-sized (portable), flexible, customizable, reconfigurable and reprogrammable with the advantages of good customization, cost-effectiveness, integration, accessibility and expandability [11]. Moreover, an FPGA can accelerate the sensor calculations due to the architecture of this device. In this way, FPGA technology offers extremely high-performance signal processing and conditioning capabilities through parallelism based on slices and arithmetic circuits and highly flexible interconnection possibilities [12]. Furthermore, FPGA technology is an alternative to custom ICs (integrated circuits) for implementing logic. Custom integrated circuits (ASICS) are expensive to develop, while generating time-to-market delays because of the prohibitive design time. Thanks to computer-aided design tools, FPGA circuits can be implemented in a relatively short space of time [13]. FPGA technology features are an important consideration in sensor applications nowadays. Recent examples of sensor developments using FPGAs are the works of Rodriguez-Donate *et al*. [14], Moreno-Tapia *et al*. [15], Trejo-Hernandez *et al*. [16] and Zhang *et al*. [17].

In this sense, the main objective of this work is to select an efficient belief propagation algorithm and then to implement it over a FPGA platform, paving the way for accomplishing the computational requirements of real-time processing and size requirements of the CAFADIS camera. The fast and specialized hardware implementation of the belief propagation algorithm was carried out and successfully compared with other existing implementations of the same algorithm based on FPGA.

The rest of the paper is structured as follows: we will start by describing the belief propagation algorithm. Then, Section 3 describes the design of the architecture. Section 4 explains the obtained results and, finally, the conclusions and future work are presented.

## 2. Belief Propagation Algorithm

The belief propagation algorithm [1] is used to optimize an energy function in a MRF framework. The energy function *E* is composed of a data term *E_d_* and a smoothness term *E_s_*, *E = E_d_* *+ λE_s_*, where the parameter *λ* measures the relative importance of each term. The data term is the sum of the per-pixel data costs, *E_d_* *= Σ_p_* *c_p_(d)*, where, in this case, *c_p_(d)* is the focus measure taken from the set of photographs focused at different depths that was previously synthesized. The smoothness term is based on the 4-connected neighbors of each pixel and can be written as *E_s_* *= Σ_p,q_* *V_pq_(d_p_, d_q_)* where ***p*** and ***q*** are two neighboring pixels. Although there exist other ways to define *V_pq_(d_p_, d_q_)*, here the following definition was used:

(1) $$V_{\text{pq}}(d_{p},\;d_{q})=\{\begin{array}{ll} 0 & \mathrm{if}\;d_{p}=d_{q}\\ 1 & \text{otherwise} \end{array}$$

The energy function is optimized using an iterative message passing scheme that passes messages over the 4-connected neighbors of each pixel in the image grid. Each message consists in a vector of *k* positions, one for each depth level taken into account, and is computed using the following update rule:

(2) $$M_{p\to q}^{i}(d_{q})=\underset{d_{p}}{\text{min}}\left(c_{p}(d_{p})+\mu \sum_{s\in N(p)}M_{s\to p}^{i-1}(d_{p})-M_{q\to p}^{i-1}(d_{p})+\lambda \cdot V_{\text{pq}}(d_{p},d_{q})\right)$$

where $M_{p\to q}^{i}(d_{q})$ is the message passed from pixel ***p*** to pixel ***q*** for depth level *d_q_* at iteration *I*, *N*(***p****)* is the four-connected neighborhood of pixel ***p*** and *μ ∈* (*0,1*].

After a certain number of iterations *I*, the algorithm is expected to converge to the solution. Then the belief vector for every pixel has to be computed to obtain the depth level at which each pixel is focused and, finally, the depth at which the object that images on that pixel is located. The belief vector for pixel ***q*** is computed as follows:

(3) $$b_{q}(d_{q})=c_{q}(d_{q})+\mu \sum_{p\in N(q)}M_{q\to p}^{I}(d_{q})$$

The depth value for pixel ***q*** is the depth level *d_q_* with minimum belief value. This general approach of the message passing rule requires *O(k^2^* *n I)* execution time, where *k* is the number of depth levels, *n* is the number of pixels in the image and *I* is the number of iterations. Notice that the message for each pixel could be computed in parallel taking *O(k^2^)* time for each iteration. Using the techniques described in [1], the timing requirements and arithmetic resources can be reduced drastically. This is a benefit for implementing the algorithm on FPGA, since less of the valuable resources of the FPGA will be necessary for each pixel.

Two of the approaches used in [1] in order to save computation time and memory are to transform the quadratic update rule into a linear update rule taking into account the particular structure of *V_pq_(d_p_, d_q_)* and to use a bipartite graph approach in order to perform the computations in place and in half the time.

The transformation of the general message update rule gives the following update rule:

(4) $$M_{p\to q}^{i}(d_{q})=\text{min}\left(h_{p\to q}(d_{q}),\underset{d_{p}}{\text{min}}\left(h_{p\to q}(d_{p})+\lambda \right)\right)$$

with:

(5) $$h_{p\to q}(d_{p})=c_{p}(d_{p})+\mu \sum_{s\in N(p)}M_{s\to p}^{i-1}(d_{p})-M_{q\to p}^{i-1}(d_{p})$$

This allows computation of the message update for each pixel in *O(k)* time and allows a saving in arithmetic resources.

On the other hand, one can observe that the image grid can be split into two sets so that the outgoing messages of a pixel in set A only depends on the incoming messages from neighbors in set B, and *vice-versa*. This gives a checkerboard-like pattern, where the message updating rule is computed at odd iterations for pixels in set A and at even iterations for pixels in set B. With this approach, all messages are initialized at zero and the updating is then alternatively conducted on the two sets. Once the algorithm converges to the solution, the belief vector can be computed in the usual way, since no significant difference is expected between an iteration message and the previous one, so that $M_{p\to q}^{i}(d_{q})\approx \;M_{p\to q}^{i-1}(d_{q})$. With this technique the memory requirements are reduced to half and computing speed is doubled.

## 3. Algorithm to Hardware

**Figure 1.**
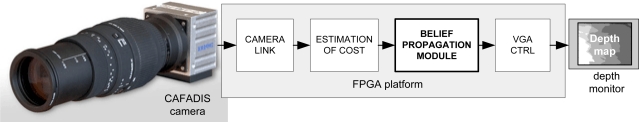
Overall system to be integrated in a portable video camera.

The global control system to be developed is shown in Figure 1. The functional architecture has four sub-modules. At the front of the system the camera link module receives data from CAFADIS in a serial mode. The following stages perform the digital data processing using FPGA resources. The second and the third stages are the estimation of cost and the belief propagation algorithm respectively. The estimation of cost is a less demanding computation and the main computing power is carried out by the belief propagation. Finally, a simple VGA controller is necessary to display the depth data.

We will focus on the FPGA implementation from Equations 4 and 5 to improve processing time. The pseudo-code for the belief propagation algorithm is as follows.

The algorithm can be accelerated using parallel processing power of FPGAs instead of other classical technology platforms. In our implementation the improvements are due to the fact that:

- Arithmetic computations are performed in pipeline and as parallel as possible.
- The number of planes in the architecture implemented is parallelized.

**Figure 2.**
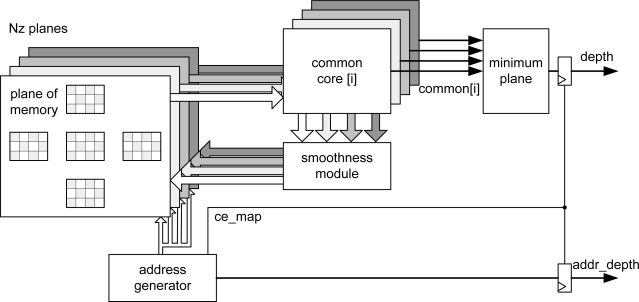
Architecture of the designed belief propagation system.

Taking into account these considerations, the overall implemented architecture is depicted in Figure 2. The address generation unit acts as the global controller of the system. In order to carry out the smoothing, the new values of messages are recalculated using the appropriate values that are extracted from the memory cost and message passing at each iteration. This phase is computed into the *common_core* module for each plane.

Finally, the smoothing module compares the new values obtained from all levels and the new values are stored in the message passing memory after smoothing (Table 1).

These steps are performed using gray pixels in odd iterations and the white pixels in even iterations (Figure 2). The number of iterations is configured in the *address generator* module.

Simultaneously, the *common core* calculates the common part of the four outgoing messages for each plane. Then the incoming message from each neighbor in the previous iteration is subtracted from the common part. After that, the minimum value for each direction is computed and smoothed in the smoothing module. Finally, the belief function is computed in the *minimum plane* block in order to select the plane to which each pixel belongs. When the address generation module completes its iterations, it enables the output of this block, providing the distance associated to each pixel as output signal. These data are obtained alternately (even and odd pixels) in line with the inherent addressing of the algorithm to minimize resources and execution time. The distance data can be flipped using a double dual-port memory system at the output of the *minimum plane* module preserving the pipeline [18,19].

The implementations of each of the modules that make up the overall architecture are detailed below.

### 3.1. Memory planes

According to the algorithm, each memory plane consists of one cost memory and four message-passing memories.

**Figure 3.**
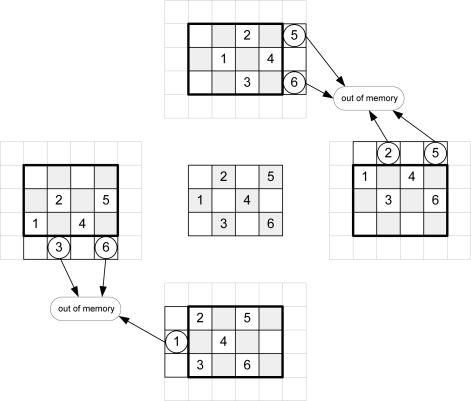
Memory addressing for even iterations.

**Figure 4.**
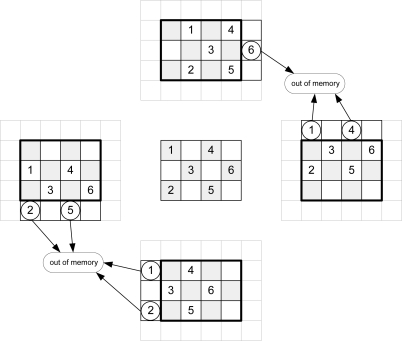
Memory addressing for odd iterations.

Taking into account Equation 1, Figure 3 shows the addresses of message-passing memory which must be accessed in order to perform the arithmetic computation for an image of Nx = 3 and Ny = 4 pixels for even iterations. Figure 4 depicts the same considerations for odd iterations.

To calculate the new messages associated with a given pixel, the up-memory must supply the value of its right, the down-memory, the value of the left, and the left and right-memories should access the top and bottom positions respectively. This addressing causes conflicts at the ends of the arrays. In Figure 3, for example, in order to compute the calculations for pixel 1, the down-memory address is out of range. Table 2 shows the memory accesses to be performed for the example in Figures 3 and 4.

The software algorithms solve these conflicts using zero padding. This implies an extra memory of 8Nx + 8Ny + 16 for each plane in a hardware implementation. A second approach is to avoid this zero padding. As shown in Figures 3 and 4, each message memory only has conflicts on one side of the array. Taking this into account, the extra memory used is reduced to 2Nx + 2Ny for each **Nz** plane.

However, the FPGA's internal memory is a critical resource when implementing this algorithm and the final design optimizes the memory usage by eliminating the above mentioned excesses. Instead of increasing memory sizes, additional logic was added in the address generator design in order to indicate when an address is valid. With this alternative design, the size of the memory is minimized. Furthermore, the size is the same for all the memories, making the VHDL implementation more modular and flexible.

### 3.2. Address generator

**Figure 5.**
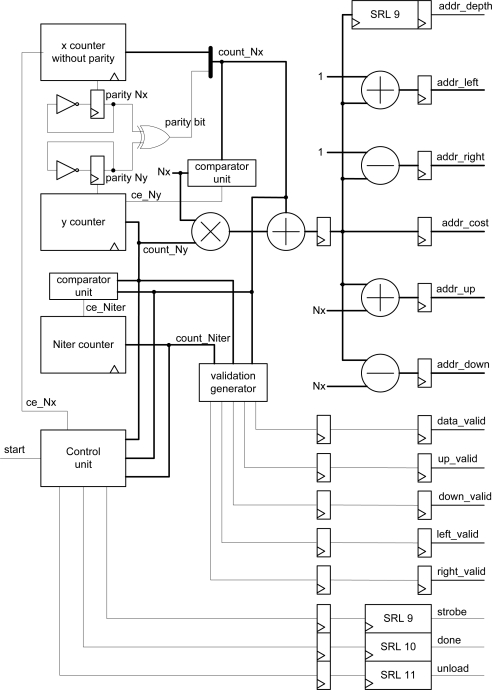
Architectural block diagram of the address generator.

The block diagram of the address generator and control signals are depicted in Figure 5. Basically, this module consists of counters, comparators, shift registers, one multiplier, three adders, two subtracters and a state machine that acts as a control unit.

The operation of the module is as follows: the x-counter is enabled when the *start* signal goes high. The property of this counter is that it lacks the least significant bit index, whose value is calculated with a parity circuit to implement the checkerboard algorithm.

The effective address is generated using *count_Nx* and *count_Ny* counters. This value is calculated by multiplying the number of rows (**Nx**) by *count_Ny* and then adding the current value of the row (*count_Nx*). Based on the current cost address, the values of the message addresses are easily obtained, as well as the address of the plane corresponding to the value calculated (delay of nine clock cycles to synchronize with the arithmetic module). Furthermore, when the values of *count_Ny* and *count_Nx* reach the maximum value, the next iteration is enabled through a third counter (*count_Niter*). The v*alidation_generator* block uses these three values to estimate if the message addresses are valid.

The control unit provides *strobe*, *unload* and *done* signals that are estimated using the value of the three counters. These signals are passed by shift registers to preserve the overall system synchronization.

The validity of the message addresses can be calculated using only the *count_Nx* and *count_Ny* pixel counters (see Figures 3, 4). However, the inclusion of the iteration counter saves on resources and execution time of the algorithm. In fact, when the algorithm achieves the last iteration for an image, message memories contain values that are not valid for the next image. The message memory must be empty at the first iteration for a given frame. This implies the use of two sets of memories continuously commuting between odd and even frames or the implementation of an erase phase consuming extra time. Both options use more resources of the FPGA hardware. Thus, the *count_Niter* counter is included in the estimator and when the value of this counter is zero or one, this module assumes that all addresses are invalid.

### 3.3. Arithmetic module

**Figure 6.**
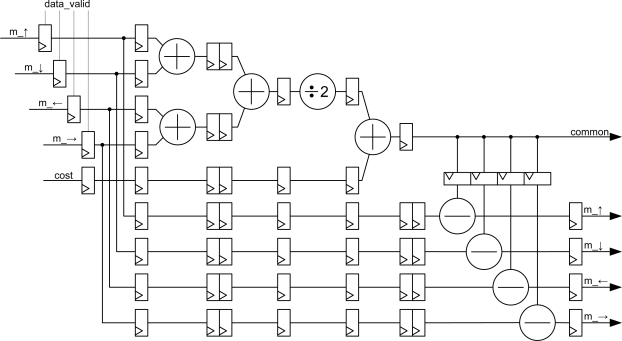
Architectural block diagram of the arithmetic core.

This module is responsible for performing the calculations of the message passing algorithm according to the equations. The implemented module is depicted in Figure 6. Several registers are included in the circuit to perform the computation in pipeline. *Data_valid* signals are connected to the synchronous reset of the first registers by passing the data, so that if the data are invalid, the values at which operations are carried out are zero.

The value of the *common* signal is calculated using current messages and the value of the *cost* signal as shown at the top of the figure. Simultaneously, message data are delayed so that they can be subtracted from the value of the *common* signal at the bottom of the figure. This architecture preserves the pipeline feature and the FPGA only needs nine clock cycles to carry out the computation of the new message data in parallel mode. The pipeline continuously provides new data after the latency time.

Intermediate values of the arithmetic module are conveniently rounded. So, the input precision is the same as the output precision (generic *data_width*).

The module is synthesized **Nz** times (depending on the number of planes, see Figure 2).

### 3.4. Smoothness unit

**Figure 7.**
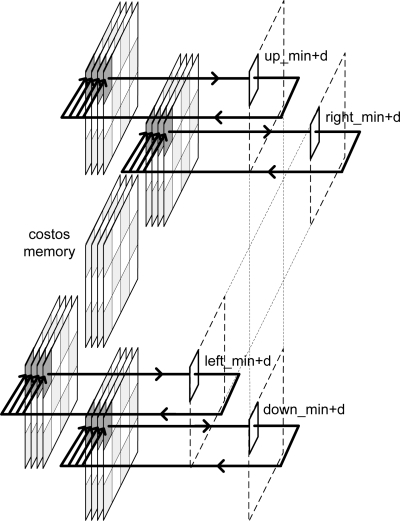
Diagram of the smoothing operation.

This module performs the smoothness corresponding to the last line of the pseudo-code of Table 1. At first, it calculates the minimum of messages for all planes with a comparator tree. Then, a constant factor (**d**) is added and the result is compared to the estimated value for each memory. Finally, the minimum between these two values is stored in the memory (Figure 7). Only 2 + ⌈log_2_ *Nz*⌉ clock cycles are necessary for this operation.

### 3.5. Depth estimator

This block selects the plane that contains the minimum of *common* values in each cycle. Note that the result is only enabled when all the iterations have been carried out. This feature is controlled through a *ce_map* signal. This signal is internally connected with the strobe signal of the arithmetic module. This module requires two clock cycles.

### 3.6. Size considerations of the design

The update of the messages takes 13 clock cycles (9 from the arithmetic and 2 + ⌈log_2_ *Nz*⌉ from the smoothness module). Thus, we must have:

(6) $$\left\lfloor \frac{Nx\cdot Ny}{2}\right\rfloor -\left\lfloor \frac{Nx}{2}\right\rfloor >11+\left\lceil {\text{log}}_{2}Nz\right\rceil$$

since *addr_down = addr – Nx* is the worst case in the address generator module (Figure 5). The divisions by 2 are due to checkerboard optimization. If this equation is not satisfied, a non-updated value from memory is read. This means that the frame must be 8 × 8 or higher.

## 4. Results

**Figure 8.**
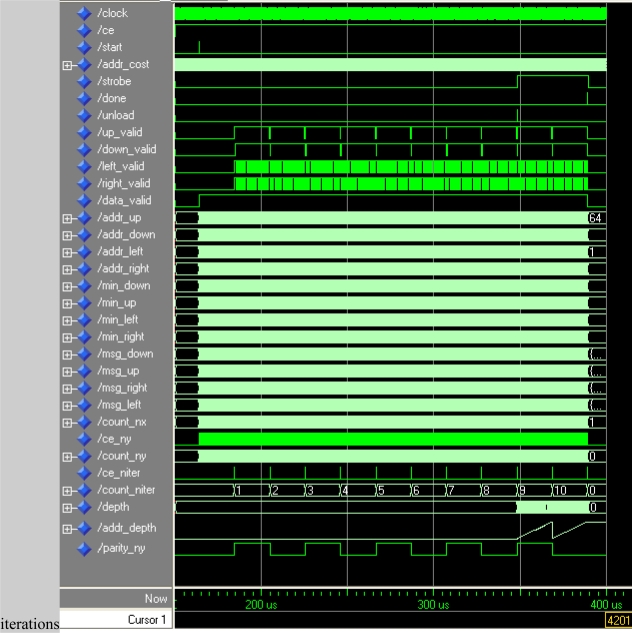
Functional simulation of belief propagation for a 64 × 64 frame and 10.

A first script was successfully tested using Matlab. Then the design was programmed using the VHDL hardware description language, simulated using ModelSim, and XST was used to synthesize these modules. An overview of the module operation is shown in a functional simulation (Figure 8).

**Figure 9.**
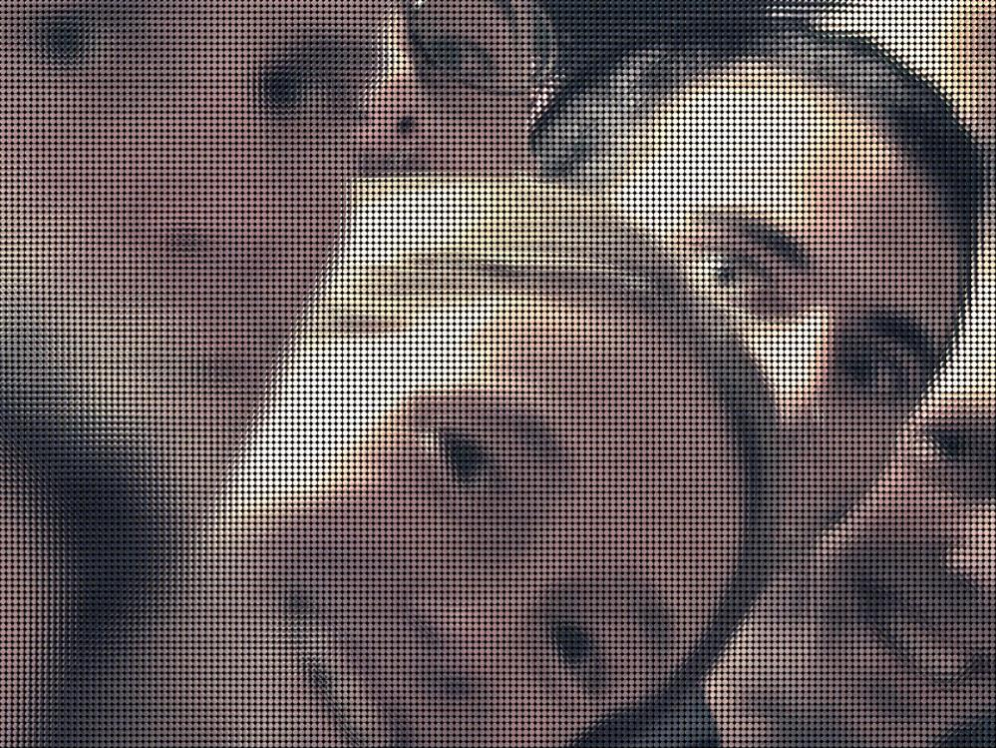
Lightfield captured with a plenoptic camera. Image taken from [5].

**Figure 10.**
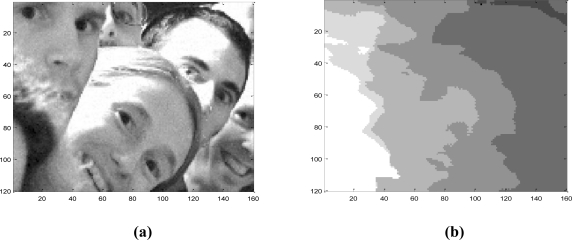
**(a)** Single image. **(b)** Depth map.

Figure 9 depicts the original plenoptic image used for simulations. In figure 10 the final depth map of a test image is displayed together with the associated single image. The belief propagation prototype was synthesized with a Xilinx XC5VSX50T Virtex-5. This FPGA is provided in a ML506 Xtreme DSP development platform. This development board has a 200 MHz clock.

The depth estimation using multistereo is less clear than using stereo because the cost function is more complex. Moreover, the quantifying results with 16 bit precision have shown performances really close to the original Matlab programmed algorithm.

### 4.1. Time analysis

The implemented architecture is pipeline and it permits continuous data streaming. The use of internal memory allows simultaneous accesses to the messages for each direction and each plane. Also, all arithmetic computations have been replicated for each plane and the number of cycles in order to make the final depth map independent of the number of planes. Taking into account this and the checkerboard algorithm, the cycles for the operation of the module are:

(7) $$\mathrm{cycles}=\frac{\mathrm{Nx}\cdot \mathrm{Ny}}{2}\cdot \left(\mathrm{Niter}+1\right)+\left\lceil {\text{log}}_{2}\mathrm{Nx}\right\rceil +9\approx \frac{\mathrm{Nx}\cdot \mathrm{Ny}}{2}\cdot \left(\mathrm{Niter}+1\right)$$

Table 3 shows the cycles and the total time broken down into the stages of the total system for several **Nx**, **Ny** and **Niter** values. A 200 MHz clock has been assumed.

These results can be contrasted with other works. [20] and [21] have proposed FPGA-based implementation of belief-propagation algorithm for stereo vision instead of multistereo vision. Their implementations use external memory and the system latency is mainly limited by the memory accesses. Their algorithms produce good results but the computation is slow and the 3D reconstruction is not possible in real-time. For example, in [20] authors obtain a depth map in 0.32 s for 1,280 × 720 pixels using a Virtex-5. Our architecture based on internal memory reduces the time needed to calculate the depth estimation map by approximately 10 times with respect to an external memory implementation.

### 4.2. Area

Block RAMs are the critical resource for the implementation of the system in a FPGA device. Table 4 shows the memory resources used for several FPGAs with 16-bit precision. Other resources such as DSP48 or slices are always below 10% for the FPGAs under consideration.

## 5. Conclusions and Future Work

The current investigation develops a first FPGA implementation for depth map estimation using the belief propagation algorithm for the CAFADIS plenoptic sensor. The main contribution of this work is the use of FPGA technology for processing the huge amount of data from the plenoptic sensor. FPGA technology features are an important consideration in the CAFADIS camera. The depth reconstruction in real time is ensured due to the extremely high-performance signal processing and conditioning capabilities through parallelism based on FPGA slices and arithmetic circuits and highly flexible interconnection possibilities. Furthermore, the use of a single FPGA can meet the size requirements for a portable video camera. The low cost of FPGA implementation in data processing makes the camera sellable at not too expensive prices in the future.

However, algorithm implementation requires an extremely large internal memory. Such massive amount of storage requirement becomes one of the most crucial limitations for the implementation of Virtex-4, Virtex-5 and Virtex-6 FPGA families and the development platform has to be replaced by a subsequent generation of FPGA. The quantifying results with 16 bit precision have shown performances are really close to the original Matlab programmed algorithm. Our results have been compared with other belief propagation algorithms in FPGA and our implementation is comparatively faster.

The design of the belief algorithm was developed using functional VHDL hardware description language and is technology-independent. So, the system can be implemented on any large enough FPGA. Xilinx has just announced the release of 28-nm Virtex-7 FPGAs. These devices provide the highest performance and capacity for FPGAs (up to 65Mb) [22,23] and they will allow algorithm implementation for larger images.

In the future, we will implement this architecture in a Virtex-7 and integrate it in a real-time multistereo vision system. The goal is to obtain a fully portable system.

## Figures and Tables

**Table 1.** Pseudo-code for the algorithm.

**for** z = 1:Nz msg_min = inf; **for** n=1:Niter **for** y=2:Ny+1 offset = rem(y+n,2); **for** x=2+offset:2:Nx+1 % Applying checker board common = cost(z,x−1,y−1) + mu * (msg(z,x,y1,↓) + msg(z,x,y+1,↑) + + msg(z,x−1,y,→) + msg(z,x+1,y,←)); % Update messages msg(z,x,y,↑) = common − msg(z,x,y−1,↓); msg(z,x,y,↓) = common − msg(z,x,y+1,↑); msg(z,x,y,←) = common − msg(z,x−1,y,→); msg(z,x,y,→) = common − msg(z,x+1,y,←); msg_min = min(msg_min,msg(z,x,y,:)) **end**; **end**; **end**; % Applying smoothing. msg(z,x,y,:) = min(msg_min+d, msg(z,x,y,:)); **end**;

Nx and Ny determine the size of the image, and Nz is the number of planes.

**Table 2.** Address generation for the example.

**Iteration**	**Cost address**	**Up**	**Down**	**Left**	**Right**
odd	0	3	out	1	out
even	1	4	out	2	0
odd	2	5	out	out	1
even	3	6	0	4	out
odd	4	7	1	5	3
even	5	8	2	out	4
odd	6	9	3	7	out
even	7	10	4	8	6
odd	8	11	5	out	7
even	9	out	6	10	out
odd	10	out	7	11	9
even	11	out	8	out	10

**Table 3.** Execution time for the belief algorithm in FPGA.

**Nx**	**Ny**	**Niter**	**Cycles**	**Time [ms]**
64	64	10	22,539	0.11
64	64	25	53,259	0.27
120	160	10	105,611	0.53
120	160	25	249,611	1.25
128	128	10	90,123	0.45
128	128	25	213,003	1.07
256	256	10	360,459	1.80
256	256	25	851,979	4.26
512	512	10	1,441,803	7.21
512	512	25	3,407,883	17.04
1,024	1,024	10	5,767,179	28.84
1,024	1,024	25	13,631,499	68.16

**Table 4.** FPGA internal memory resources.

**FPGA device**	**Configuration of image**	**Basic internal RAM module**	**BRAM (used/available)**
XC4SX35 Virtex-4	64 × 64 × 4	RAMB16 1K × 16	80/192 (41%)
XC5SX50 Virtex-5	64 × 64 × 4	BRAM 2K × 16	40/132 (30%)
XC5SX50 Virtex-5	64 × 64 × 8	BRAM 2K × 16	80/132 (60%)
XC6VLX240 Virtex-6	64 × 64 × 8	BRAM 2K × 16	40/416 (9%)
XC6VLX240 Virtex-6	64 × 64 × 8	BRAM 2K × 16	80/416 (19%)
XC6VLX240 Virtex-6	128 × 128 × 4	BRAM 2K × 16	160/416 (38%)
XC6VLX240 Virtex-6	128 × 128 × 8	BRAM 2K × 16	320/416 (77%)
XC6VLX240 Virtex-6	256 × 128 × 4	BRAM 2K × 16	320/416 (77%)
